# Coronavirus Disease 2019 Pneumonia and Acute Pancreatitis in a Young Girl

**DOI:** 10.7759/cureus.15374

**Published:** 2021-06-01

**Authors:** Paghunda Ehsan, Muhammad Haseeb, Zaraq Khan, Aiman Rehan, Romil Singh

**Affiliations:** 1 Medicine, Hayatabad Medical Complex Peshawar, Peshawar, PAK; 2 Internal Medicine, Jinnah Hospital Lahore, Lahore, PAK; 3 Internal Medicine, Hayatabad Medical Complex Peshawar, Peshawar, PAK; 4 Internal Medicine, Dow University of Health Sciences, Karachi, PAK; 5 Critical Care, Mayo Clinic, Rochester, USA

**Keywords:** covid-19, sars-cov-2, pneumonia, acute pancreatitis, coronavirus disease 2019

## Abstract

Severe acute respiratory syndrome coronavirus 2 pneumonia and acute pancreatitis are rarely reported in patients with coronavirus disease 2019 (COVID-19). We present the case of a 13-year-old girl who presented with nausea, vomiting, and abdominal pain for the last two days, along with a cough for the last week. She had a fever and tachycardia. Lung examination revealed reduced breath sounds, and abdominal examination showed tenderness in the epigastrium. COVID-19 polymerase chain reaction was positive, and her serum chemistry revealed elevated serum amylase and lipase. Abdominal computed tomography revealed diffuse inflammation of the pancreas with peripancreatic edema, and chest X-ray demonstrated diffuse infiltrates and pneumonic patches in both lungs. Her initial management included bowel rest, intravenous fluids, intravenous remdesivir, and azithromycin with supplemental oxygen based on the provisional diagnosis of COVID-19 pneumonia and acute pancreatitis. Her abdominal symptoms started improving, and dexamethasone was added to her regimen due to her worsened respiratory condition. She was symptom-free on day seven except for a mild cough. She was discharged on day eight with follow-up.

## Introduction

Coronavirus disease 2019 (COVID-19) has spread rapidly around the world, causing a pandemic and affecting individuals of all ages [1,2]. A study reported that around 4% of COVID-19 patients were below the age of 18 in the United States [3]. COVID-19 has a typical manifestation of the respiratory system and generally presents with fever, cough, dyspnea, and fatigue. COVID-19 has also demonstrated signs and symptoms in the gastrointestinal (GI) system, cardiovascular system, and neurological system [4-7]. Nausea, vomiting, abdominal pain, and diarrhea have also been reported as an initial manifestation of severe acute respiratory syndrome coronavirus 2 (SARS-CoV-2) [8]. Acute pancreatitis induced by COVID-19 pneumonia in children is rarely reported in the literature [9]. Herein, we describe a case of acute pancreatitis associated with COVID-19 pneumonia in a young girl.

## Case presentation

A 13-year-old girl without a previous medical problem was brought to the emergency department for nausea, persistent nonbloody vomiting, and abdominal pain for the last two days, along with one episode of loose stool. Abdominal pain was sudden in onset, progressive, and located in the epigastrium. She also had a mild cough and fever for the last week. She was previously healthy with no history of fever and ill contacts. Initial evaluation revealed a temperature of 100°F, tachypnea (31/minute), tachycardia (103/minute), and borderline oxygen saturation (93%). On clinical examination, she looked ill, and her lungs were clear with reduced breath sounds in the lower lobe of the right lung. Abdominal examination was unremarkable except for tenderness in the epigastrium. A nasal swab for COVID-19 polymerase chain reaction was done as part of the regular screening which was positive. Initial laboratory investigations revealed elevated serum lipase, amylase, erythrocyte sedimentation rate (ESR), and C-reactive protein (CRP) (Table 1).

**Table 1:** Initial blood workup.

Parameter	Day 1	Day 6	Reference range
White blood cells	10,007	8,899	4,000-11,000/mm^3^
Red blood cells	5.1	5.0	4.35-5.65 million cells/mm^3^
Hemoglobin	12	12	12-15.5 mg/dL
Aspartate aminotransferase	46	37	8-35 IU/L
Alanine aminotransferase	44	41	7-35 IU/L
Serum lipase	2,331	877	0-160 IU/L
Serum amylase	598	189	30-110 IU/L
Serum triglyceride	135	134	<150 mg/dL
Serum creatinine	1.0	0.9	0.7-1.2 mg/dL
Blood urea nitrogen	19	20	8-20 mg/dL
Lactate dehydrogenase	419	295	140-280 IU/L
Erythrocyte sedimentation rate	37	23	<22
C-reactive protein	14	04	<0.2 mg/dL
Total bilirubin	1.5	1.3	0.3-1.2 mg/dL
D-dimer	1,431	487	<250 ng/L
Creatine kinase	202	139	30-135 IU/L

**Figure 1:**
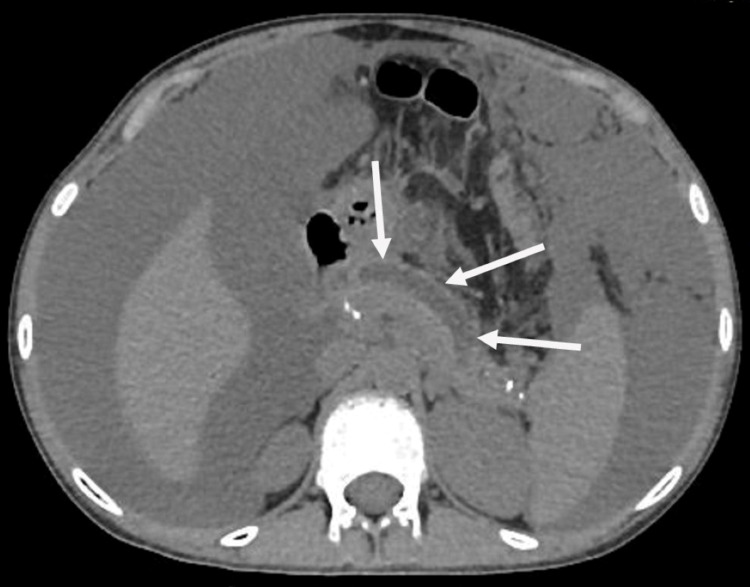
CT abdomen showing pancreatic inflammation and edema (white arrows). CT: computed tomography

**Figure 2:**
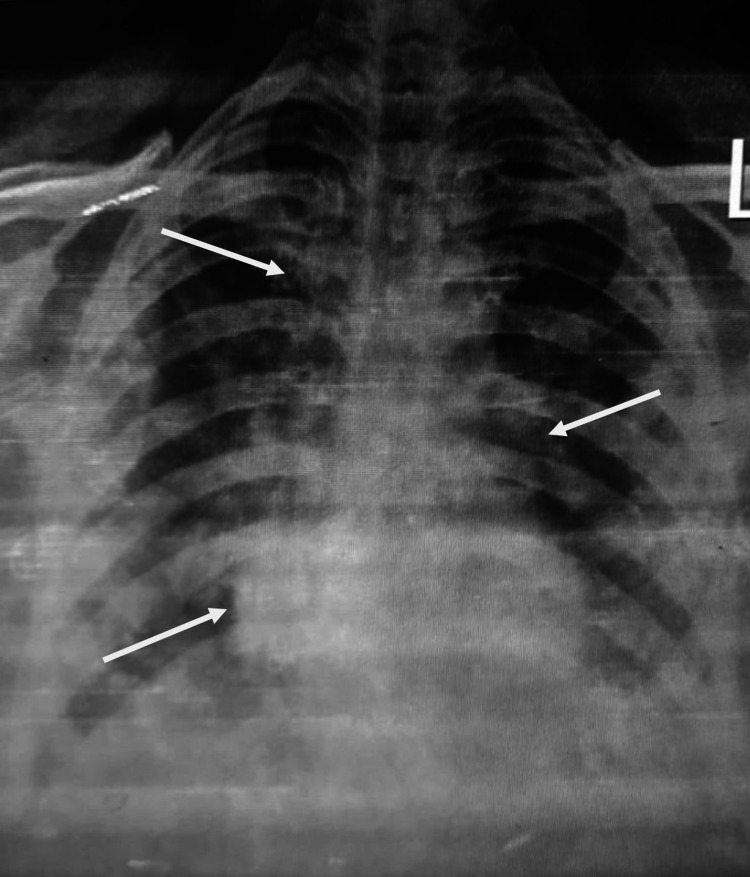
Chest X-ray showing infiltrates and pneumonic patches in both lungs.

Abdominal ultrasonography was nonsignificant with no evidence of gallstone, and the pancreas was not visualized. Abdominal computed tomography (CT) was performed, which revealed diffuse inflammation of the pancreas with peripancreatic edema without necrosis, consistent with acute pancreatitis (Figure 1). Her chest X-ray demonstrated diffuse infiltrates and pneumonic patches in the lower lobe of both lungs (Figure 2).

Diagnostic workup for infection, including blood culture and sputum culture, were negative. She had no history of previous pancreatitis and no recognized risk factors such as family history, trauma, gallstone, alcohol consumption, or medication exposure. Due to the lack of any identifiable risk factor, she was diagnosed with acute pancreatitis and pneumonia due to COVID-19. She was admitted to the gastroenterology ward to treat acute pancreatitis, and infectious disease consultation was taken to manage COVID-19 pneumonia.

Her initial management included bowel rest, dietary restrictions, intravenous fluids, antiemetics, and analgesics. For COVID-19 pneumonia, she was commenced on intravenous remdesivir (5 mg/kg) and azithromycin (20 mg/kg) with supplemental oxygen. Her abdominal symptoms started improving on day two; however, her respiratory system worsened with a gradual drop in oxygen saturation to 85%. She was shifted to the intensive care unit for aggressive management. She was commenced on dexamethasone (0.15 mg/kg) in addition to azithromycin and remdesivir, and she required oxygen at 3 L/minute by nasal cannula. She was stable on day four of hospitalization and was symptom-free on day six except for a mild cough. She received seven days of treatment and was discharged on day eight with follow-up. She was doing well on her recent visit seven days later. Her laboratory investigations on day one and day six are shown in Table 1.

## Discussion

Acute pancreatitis is a medical emergency requiring immediate diagnosis and resuscitation, with a mortality rate up to 30% in severe cases. Multiple conditions can induce acute pancreatitis. Alcohol consumption, gall stone, and hypertriglyceridemia are among the common causes of acute pancreatitis [10]. Many viruses have also been implicated as causative agents of acute pancreatitis. However, COVID-19-induced pancreatitis is not reported widely in the literature, and only a small number of cases have been reported. A study from China underlined that 17% of COVID-19 cases had elevated pancreatic enzymes, and a case series of three COVID-19 patients described acute pancreatitis in two cases [11,12]. Kataria et al. reported a case of acute pancreatitis as an initial manifestation of COVID-19 [9]. Our case involved a 13-year-old girl who presented with vomiting, abdominal pain, dry cough, and fever, which was diagnosed as acute pancreatitis and COVID-19 pneumonia.

The mechanism of acute pancreatitis due to COVID-19 is not yet defined. COVID-19 is an inflammatory disease characterized by a widespread immune response throughout the body. Angiotensin-converting enzyme 2 (ACE2) receptors are responsible for attachment and invasion inside the human cells [13,14]. These receptors are predominately expressed in alveolar cells of the lungs, epithelial lining of the GI tract, and pancreatic islets, responsible for GI manifestation and elevation of pancreatic enzymes in COVID-19 patients, along with respiratory manifestations [15]. Possible mechanisms have been explained for COVID-19-induced pancreatitis. SARS-CoV-2 may directly cause acute pancreatitis due to the higher expression of ACE2 receptors in pancreatic islets. Acute pancreatitis can be secondary to intense inflammatory response and cytokine storm induced by COVID-19 infection [16,17]. In our case, the patient had laboratory evidence of marked systemic inflammation with increased ESR, CRP, and D-dimer.

Diagnosis of acute pancreatitis is by clinical, serology, and imaging modalities. Serum amylase and lipase are highly specific, and three times greater than the standard value is considered diagnostic. Ultrasound and CT of the abdomen help in diagnosis and ruling any other complication of acute pancreatitis. Management of acute pancreatitis is conservative and symptomatic [10]. Treatment involves bowel rest, resuscitation with isotonic fluids, and analgesics. The standard of care in children with acute pancreatitis includes painkillers and crystalloid intravenous fluid [18]. Recently published guidelines for critically ill patients with SARS-CoV-2 recommend the conservative fluid strategy and maintaining fluid balance [19].

## Conclusions

Our case highlights the need for an immediate evaluation of COVID-19 patients who present with GI manifestations for the possibility of acute pancreatitis. COVID-19 has a variable presentation due to the involvement of other systems and leads to poor prognosis and prolonged hospital stay if the case is not managed appropriately. Our case encourages physicians to investigate the GI manifestations carefully in patients with SARS-CoV-2 to prevent delay in management and potential complications. There is also a need to identify a possible relationship between acute pancreatitis and COVID-19.
